# The Challenges and Strategies for Cleaning the Routinely Collected Data for Total Knee Replacements (TKR) and Total Hip Replacements (THR) in the Kingdom of Fife

**DOI:** 10.23889/ijpds.v9i5.2777

**Published:** 2024-09-18

**Authors:** Luciana Pedro, Muhammad Akhtar, Arthur Winata, Colin McCowan

**Affiliations:** 1 School of Medicine, University of St Andrews; 2 Victoria Hospital Kirkcaldy, NHS Fife

Knee and hip replacement surgeries have revolutionised orthopaedic medicine by providing individuals with surgeries resulting in pain relief, improved mobility and a better quality of life. NHS Fife has been collecting routine data since 1998 on patient demographics, type of surgery, recovery time, follow-ups, revision, and outcomes, including complications. This work reports the challenges and strategies to clean the collected data.

Data was available for 12,313 patients, totalising 14,524 THR and TKR surgeries performed in NHS Fife between 1998 and 2021. The original dataset consisted of 34 unique-structured files covering distinct periods and representing five categories: primary surgery, follow-up, infection, mortality, and revision. Data wrangling was done using the programming language R, transforming the raw data into a more functional form. The main elements of the data-cleaning procedure were combining variables representing the same information and converting void/corrupted/purposeless values into missing data, providing a standardisation for the collected information. Then, a rigorous data merging resulted in reshaping this complex collection of files into one single streamlined file for each of the five categories and type of surgery. Patients who had multiple surgeries were also cross-identifying, enabling the characterisation of the epidemiology of multiple lower limb joint replacements.

The structured files can now be linked to studies across different datasets like social care, prescriptions, and primary care. It can also be used to develop future machine learning models to assess the risk of multiple primary and revision knee and hip replacements and their outcomes.

